# Network-based analysis of virulence factors for uncovering *Aeromonas veronii* pathogenesis

**DOI:** 10.1186/s12866-021-02261-8

**Published:** 2021-06-24

**Authors:** Hong Li, Xiang Ma, Yanqiong Tang, Dan Wang, Ziding Zhang, Zhu Liu

**Affiliations:** 1 School of Life Sciences, Hainan University, Haikou, China; 2 Key Laboratory for Sustainable Utilization of Tropical Bioresource, College of Tropical Crops, Hainan University, Haikou, China; 3 State Key Laboratory of Agrobiotechnology, College of Biological Sciences, China Agricultural University, Beijing, China

**Keywords:** *Aeromonas veronii*, Protein-protein interaction network, Virulence factor, Pathogenesis

## Abstract

**Background:**

*Aeromonas veronii* is a bacterial pathogen in aquaculture, which produces virulence factors to enable it colonize and evade host immune defense. Given that experimental verification of virulence factors is time-consuming and laborious, few virulence factors have been characterized. Moreover, most studies have only focused on single virulence factors, resulting in biased interpretation of the pathogenesis of *A. veronii*.

**Results:**

In this study, a PPI network at genome-wide scale for *A. veronii* was first constructed followed by prediction and mapping of virulence factors on the network. When topological characteristics were analyzed, the virulence factors had higher degree and betweenness centrality than other proteins in the network. In particular, the virulence factors tended to interact with each other and were enriched in two network modules. One of the modules mainly consisted of histidine kinases, response regulators, diguanylate cyclases and phosphodiesterases, which play important roles in two-component regulatory systems and the synthesis and degradation of cyclic-diGMP. Construction of the interspecies PPI network between *A. veronii* and its host *Oreochromis niloticus* revealed that the virulence factors interacted with homologous proteins in the host. Finally, the structures and interacting sites of the virulence factors during interaction with host proteins were predicted.

**Conclusions:**

The findings here indicate that the virulence factors probably regulate the virulence of *A. veronii* by involving in signal transduction pathway and manipulate host biological processes by mimicking and binding competitively to host proteins. Our results give more insight into the pathogenesis of *A. veronii* and provides important information for designing targeted antibacterial drugs.

**Supplementary Information:**

The online version contains supplementary material available at 10.1186/s12866-021-02261-8.

## Background

*Aeromonas veronii* is one of the main pathogenic bacteria that affect aquatic animals in freshwater and seawater [1]. Infections by *A. veronii* can result in ulcerative syndrome, hemorrhagic septicaemia and mass mortality in aquatic animals such as *Oreochromis niloticus* [2], which leads to great economic losses to aquaculture industry. Humans can also be infected by *A. veronii*, hence, it is classified among quarantine objects of water quality and food safety in some countries [3, 4]. Pathogen produced virulence factors play an important role in the pathogenic process, because they enable pathogens to adhere to and invade host cells, evade host immune defenses, and compete for nutrients [5]. Although virulence factors have been identified in many pathogens, the virulence factors in *A. veronii* remain elusive.

Virulence factors can be classified into three categories based on their subcellular localization, including cytosolic, membrane associated, and secreted virulence factors [6]. Cytosolic virulence factors promote rapid adaptation of pathogens to metabolic, physiological and morphological changes, whereas membrane associated virulence factors contribute to the adhesion and pathogen evasion of host cells. On the other hand, secreted virulence factors play more important roles, as they can be delivered from pathogen cells into host cells or host environment [7, 8], allowing them to interact with host proteins to directly participate in host biological processes. Thus, identification of virulence factors, especially secreted virulence factors, is essential for understanding the pathogenesis of *A. veronii*.

Protein-protein interaction (PPI) networks are powerful tools in predicting potential virulence factors [9, 10]. For instance, Zheng et al. accurately identified the virulence factors of six species by integrating PPI networks and known virulence factors [10]. Similarly, integration of PPI networks, known virulence factors, and Kyoto Encyclopedia of Genes and Genomes (KEGG) pathways allowed Cui et al. to identify virulence factors of three species [9]. In terms of network biology, PPI networks are also fundamental in evaluating the functional importance of proteins. Given that proteins with high degree (hubs) or betweenness centrality (bottlenecks) tend to be essential proteins encoded by essential genes [11, 12], knockout or mutation of genes encoding hubs or bottlenecks will affect many phenotypic traits or result in death. For example, the lethality rate of yeast increases about threefold after knockout of genes encoding hubs compared with those encoding non-hubs [13]. Thus, many researchers are interested in exploring the topology parameters of proteins in PPI networks. PPI networks can be analyzed at the module level [14], where a module consists of physically or functionally related proteins that are assembled together to perform a specific function. Since different modules act synergetically to fulfill cellular functions, construction of PPI networks can assist in identifying key proteins and understanding pathogenic mechanisms from a systems perspective [15]. However, *A. veronii* PPI network at genome-wide scale is still not available.

Several high-throughput experimental methods, such as yeast two-hybrid screening and tandem-affinity purification coupled with mass spectrometry, have been developed to identify large-scale PPIs [16]. Due to high cost and laborious experimental methods, only the PPI networks of some model organisms have been reported, such as *Arabidopsis thaliana* [17], *Saccharomyces cerevisiae* [18], *Caenorhabditis elegans* [19], *Drosophila melanogaster* [20], *Escherichia coli* [21], and *Homo sapiens* [22]. To complement these experimental methods, a plethora of computational methods have been developed, including the widely used interolog and domain-based methods. The interolog method is mainly based on the conservation of PPIs in different organisms [23]. Two proteins are predicted to interact in an organism if they have interacting homologs in another organism. On the other hand, the domain-based method refers to two proteins that are more likely to interact if they contain interacting domains [24]. The PPI networks of many pathogens, such as *Ustilaginoidea virens* [25] and *Phomopsis longicolla* [26], have been successfully constructed based on these two PPI inference methods. In addition, these two methods have also been successfully applied to predict host-pathogen interspecies PPIs [25, 27].

In this study, potential virulence factors of the aquatic pathogen *A. veronii* were predicted and mapped onto the PPI network. The importance of the virulence factors were first evaluated based on network topology properties. Two modules enriched by the virulence factors that played important roles in *A. veronii* infection were identified. The molecular mechanisms of pathogenicity was explored by constructing the interspecies PPI network between *A. veronii* and its host *O. niloticus*. Three-dimensional structures and interacting sites were added to the interspecies PPI network to provide more interaction details that would enhance understanding of host-pathogen interactions. Finally, key residues of the virulence factors that are involved in the interaction with different host proteins were identified. These data could be leveraged for accelerated development of new antibacterial agents.

## Results

### *A. veronii* PPI network

To construct a high coverage PPI network of *A. veronii*, the two commonly used interolog and domain-based methods were applied. With the interolog method, 13,201 *A. veronii* PPIs involving 1904 proteins were obtained. Among these, most PPIs (79.74%) were derived from the model organism *E. coli*, with only 0.47% constituting the *A. veronii* PPIs derived from *A. thaliana*. When the domain-based method was used, 8328 *A. veronii* PPIs among 1479 proteins were obtained after filtering with strict standards. Thus, a total of 21,418 *A. veronii* PPIs were predicted by the interolog and domain-based methods, involving 2494 proteins (Supplementary Table S2).

### The *A. veronii* PPI network was of acceptable reliability

**Fig. 1:**
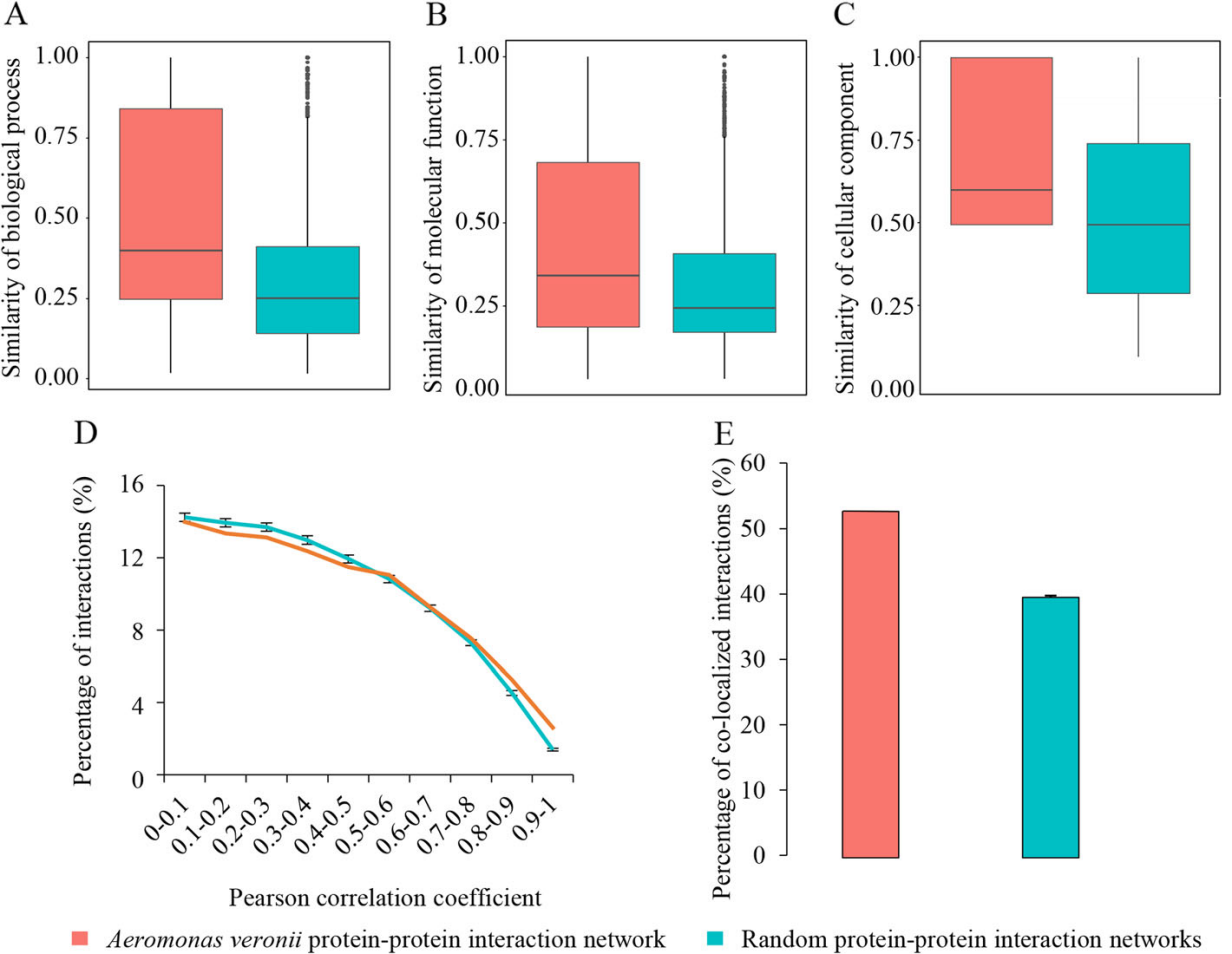
Reliability assessment of *Aeromonas veronii* protein-protein interaction (PPI) network. (A-C) Semantic similarities of gene ontology terms of interacting proteins in the *A. veronii* PPI network and one random network. The range of the box is from the first quartile to the third. The black line represents the median. The filled circle represents the outlier. (D) Percentages of interacting proteins with different Pearson correlation coefficients in the *A. veronii* PPI network and average percentages of those in 1000 random networks. (E) Percentage of co-localized interacting proteins in the *A. veronii* PPI network and average percentage of those in 1000 random networks. The error bar in (D and E) represents the standard deviation of the percentages in random networks

To evaluate the quality of the *A. veronii* PPI network, 1000 random networks were generated. Semantic similarities of Gene Ontology (GO) terms of the PPIs were first calculated. The PPIs in the *A. veronii* PPI network had significantly higher biological process, molecular function or cellular component similarities compared with those in any random network (Wilcoxon test, *p* < 2.20 × 10^− 16^; Fig. 1A-C). Specifically, 22.71% of the PPIs in the *A. veronii* PPI network had a biological process similarity of 1, whereas in the random networks, the corresponding percentage was only 5.52–6.62%. Similar results were also observed for molecular function and cellular component annotations. The percentages of PPIs sharing the same molecular function and cellular component annotations were 16.55 and 39.93% in the *A. veronii* PPI network, respectively. By contrast, the corresponding percentages in the random networks were 4.01–4.89% and 17.90–20.64%. These results indicate that the *A. veronii* PPI network is of acceptable reliability.

Similarities of gene expression patterns of PPIs were calculated based on 18 samples. The absolute Pearson correlation coefficient (PCC) values in the *A. veronii* PPI network were significantly higher than those in any random network (Wilcoxon test, *p* < 1.00 × 10^− 3^ for any random network), suggesting that the PPIs in the *A. veronii* PPI network had the tendency to be co-expressed. Although the percentages of PPIs decreased as absolute PCC values increased in both the *A. veronii* PPI network and the random networks (Fig. 1D), the random networks displayed a steeper decline when the absolute PCC value was above 0.5. Notably, at high PCC interval of 0.9–1.0, the percentage of the PPIs in the *A. veronii* PPI network was twice as much as that in the random networks (Fig. 1D). Moreover, when the percentages of PPIs with the same subcellular localization were calculated, more than 50% of the PPIs were co-localized in the *A. veronii* PPI network, whereas only 38.62–40.33% of the PPIs co-localized in the random networks (Fig. 1E). These results further indicate that the *A. veronii* PPI network is of reasonable reliability.

### Virulence factors had higher degree and betweenness centrality in the *A. veronii* PPI network

**Fig. 2:**
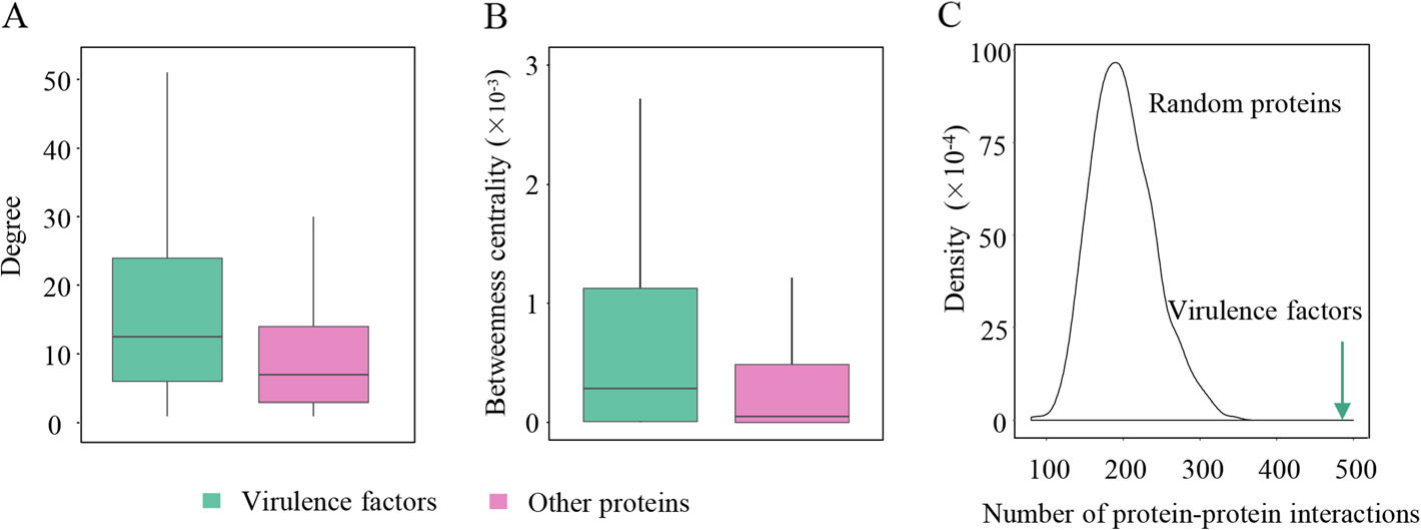
Analysis of topological properties. (A) Degree distributions of virulence factors and other proteins in the *Aeromonas veronii* protein-protein interaction (PPI) network. (B) Betweenness centrality distributions of virulence factors and other proteins in the *A. veronii* PPI network. The range of the box in (A and B) is from the first quartile to the third. The black line represents the median. (C) Statistics of the number of PPIs. The arrow points to the number of interactions formed by virulence factors. The same number of proteins as the virulence factors was randomly selected from the *A. veronii* PPI network and the number of interactions formed by the random proteins was counted. This process was repeated 1000 times. The curve represents the distribution of the number of interactions formed by the random proteins

A total of 242 potential virulence factors were predicted, of which 195 were mapped onto the *A. veronii* PPI network. When the degree and betweenness centrality were compared between the virulence factors and other proteins in the *A. veronii* PPI network, the results showed that the virulence factors had significantly higher degree and betweenness centrality than the other proteins (Wilcoxon test, *p* = 9.33 × 10^− 10^ for degree, Fig. 2A and *p* = 3.04 × 10^− 10^ for betweenness centrality, Fig. 2B). Average degree and betweenness centrality of the virulence factors were 26.75 and 3.00 × 10^− 3^, respectively. By contrast, the corresponding values for the other proteins were 16.36 and 1.00 × 10^− 3^, respectively.

### Virulence factors were enriched in two modules

**Fig. 3:**
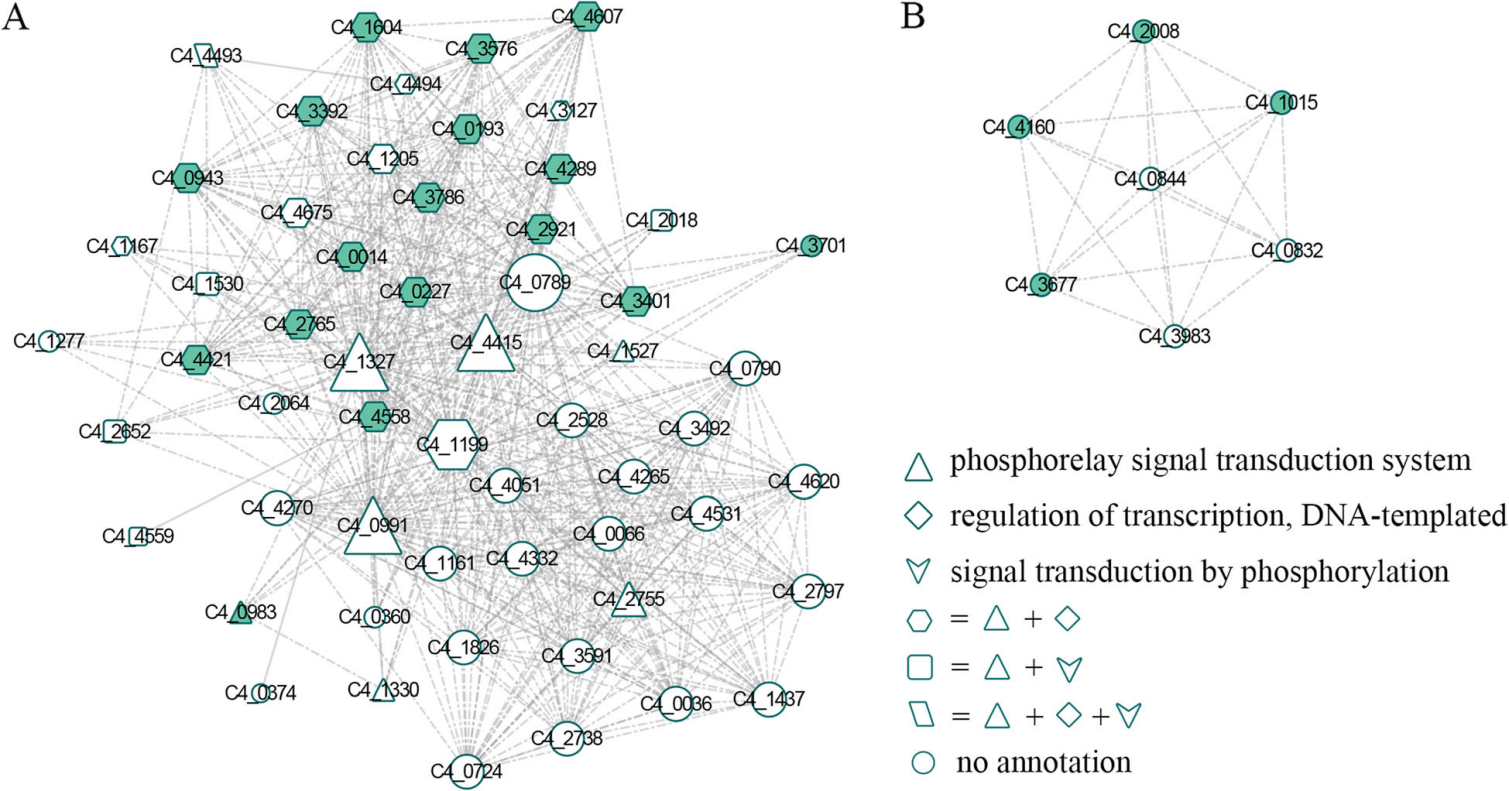
Two network modules enriched by virulence factors. The green and white nodes represent the virulence factor and the other protein, respectively. The larger node represents the protein with higher degree. The proteins with different function annotations are represented by different shapes. The solid, dash-dotted and parallel lines represent the interactions predicted by the interolog, domain-based and both methods, respectively

Although a total of 486 PPIs were formed by 195 virulence factors, when 195 proteins were randomly selected from the *A. veronii* PPI network, they formed at most 261 PPIs and at least 49 PPIs in 1000 trials (Fig. 2C), which was much less than the real number of PPIs formed by the virulence factors. These results suggest that the virulence factors have the tendency to interact, which made us speculate that the virulence factors were enriched in certain network modules. To ascertain this, the *A. veronii* PPI network was divided into 90 modules, involving 1331 proteins and 100 virulence factors. Two modules were found to be significantly enriched by the virulence factors (Fisher’s exact test, *p* = 2.36 × 10^− 7^ and 8.82 × 10^− 4^; Fig. 3).

Among the two modules, one consisted of 57 proteins, 33 of which had biological process annotations and 17 were virulence factors (Fig. 3A). This module was significantly associated with the terms “phosphorelay signal transduction system”, “regulation of transcription, DNA-templated” and “signal transduction by phosphorylation” (Fisher’s exact test, *p* = 1.80 × 10^− 38^, 1.30 × 10^− 13^ and 5.56 × 10^− 4^, respectively). Notably, 16 and 15 out of the 17 virulence factors were annotated with the terms “phosphorelay signal transduction system” and “regulation of transcription, DNA-templated”, respectively (one virulence factor was not annotated with any term). When the topology characteristics of the module in the *A. veronii* PPI network was analyzed, an average degree of the proteins in the module was 23.61, which was higher than that in the *A. veronii* PPI network (17.18). After removing the 17 virulence factors, the average degree of the proteins in the module increased (24.38). These results indicate that the module connect other modules and has a great effect on the *A. veronii* PPI network. Analysis of the other module revealed that it was enriched by the virulence factors (Fig. 3B), and consisted of seven proteins, four of which were virulence factors and could be secreted by type VI secretion system. The specific functions of these proteins in the module is however unknown.

### Virulence factors may manipulate host biological processes by mimicking and binding competitively to host proteins

**Fig. 4:**
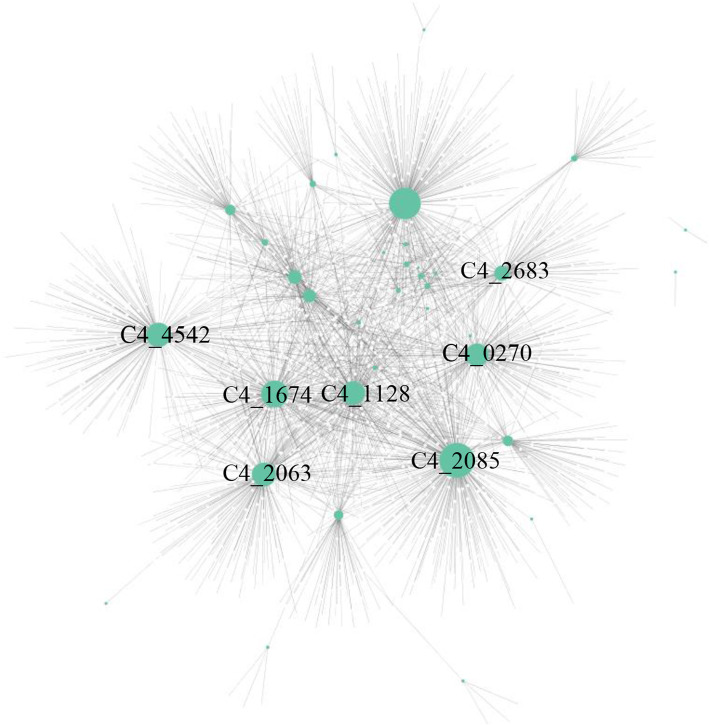
Interspecies protein-protein interaction (PPIs) between *Aeromonas veronii* and *Oreochromis niloticus.* The interspecies PPI network consisting of 36 virulence factors and 1461 *O. niloticus* proteins. The green and white nodes represent the virulence factor and the *O. niloticus* protein, respectively. The larger node represents the protein with higher degree, such as C4_2085 (succinate dehydrogenase flavoprotein subunit), C4_4642 (thioredoxin 1), C4_2063 (thioredoxin 2), C4_1128 (S-adenosylmethionine synthetase), C4_0270 (catalase), C4_2683 (ATP-dependent Clp protease proteolytic subunit) and C4_1674 (peroxiredoxin 2)

Although virulence factors could promote bacteria entry into host cells, evade or inhibit host immune responses, and obtain nutrients from hosts, it is not clear which virulence factors directly interact with host proteins. To this end, 40 (20.51%) secreted virulence factors were first predicted, out of which, 36 virulence factors were found to interact with 1461 *O. niloticus* proteins, forming 2200 interspecies PPIs (Fig. 4; Supplementary Table S3). In the interspecies PPI network, 33 virulence factors and 383 *O. niloticus* proteins had at least two partners, reflecting the complexity of interspecies PPIs. For instance, virulence factors succinate dehydrogenase flavoprotein subunit (SdhA), thioredoxin 1 (Trx1), thioredoxin 2 (Trx2), S-adenosylmethionine synthetase (MetK), catalase, ATP-dependent Clp protease proteolytic subunit (ClpP), and peroxiredoxin 2 (Prx2), had higher degree in the interspecies PPI network (Fig. 4), indicating that these virulence factors can interact with more *O. niloticus* proteins.

Many *O. niloticus* proteins, such as heat shock protein, elongation factor Tu, DNA-directed RNA polymerase subunit, Trx2, ribosomal protein S3, SdhA, peroxiredoxin 1 (Prx1), transcriptional regulator, MetK and ClpP, could interact with at least 5 proteins in *A. veronii*. In vertebrates, Trx2, MetK, ClpP, and Prx2 perform their functions by forming homo-dimers or homo-oligomers [28–31]. Moreover, our results showed that *A. veronii* Trx2, MetK, ClpP, and Prx2 were homologous and could interact with *O. niloticus* Trx2, MetK, ClpP and Prx2, respectively. These results indicate that the virulence factors mimic and bind competitively to homologous proteins in host to interfere with host biological processes.

### Structures and key interacting sites of virulence factor Trx1

**Fig. 5:**
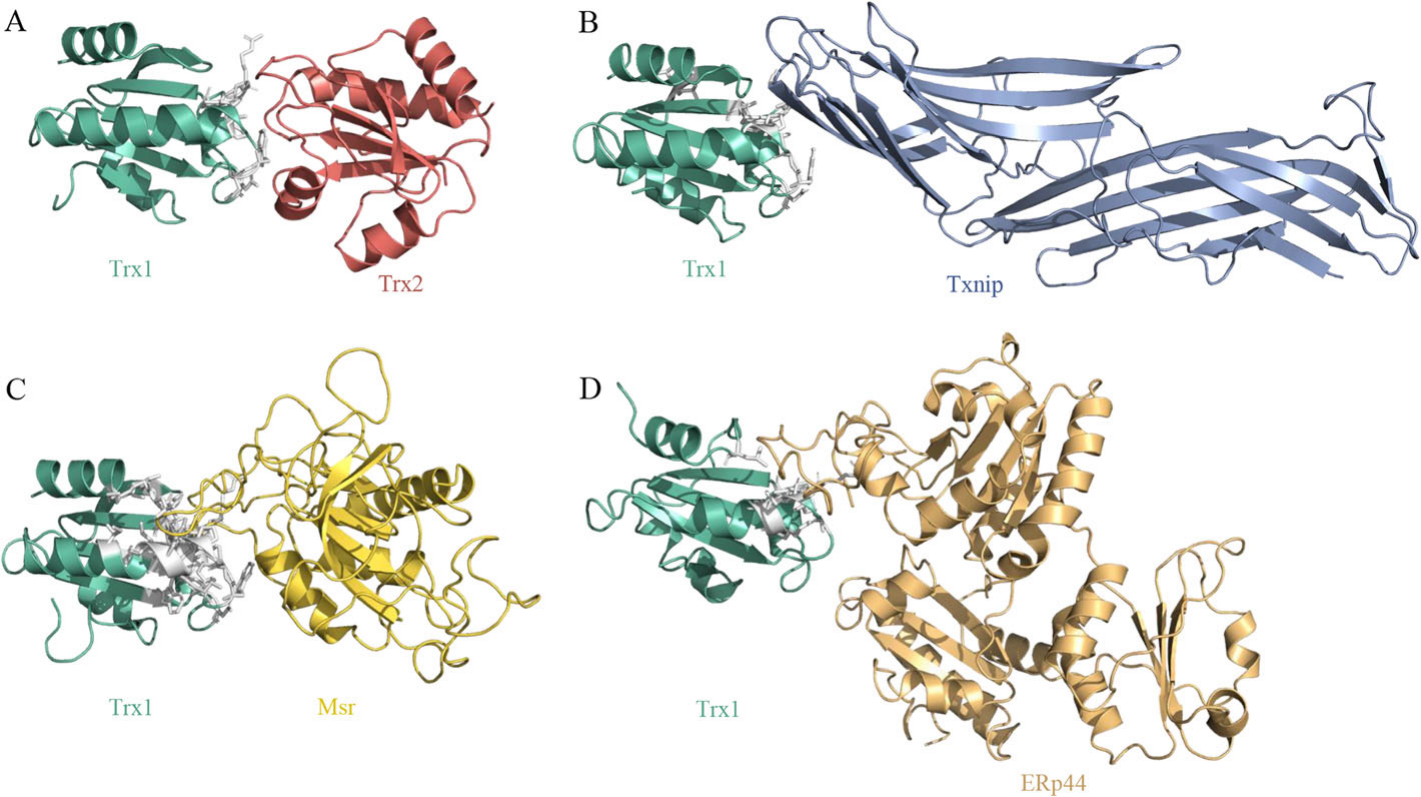
Protein complex structures formed by *Aeromonas veronii* thioredoxin 1 (Trx1) and four *Oreochromis niloticus* proteins. (A) thioredoxin 2 (Trx2), (B) thioredoxin-interacting protein (Txnip), (C) methionine sulfoxide reductase (Msr) and (D) endoplasmic reticulum resident protein 44 (ERp44). White sticks represent the interacting sites of Trx1. Trx1 binds to the four *O. niloticus* proteins using the same interface

The structures and sites of 61 interspecies PPIs formed by 15 virulence factors and 47 *O. niloticus* proteins were predicted and the data stored at https://drive.google.com/drive/folders/18cHNUOSSJ5ugmFUL1_1QLVossf5ybYUY?usp=sharing. Figure 5 shows the structures formed by the interactions between *A. veronii* Trx1 and four *O. niloticus* proteins, including Trx2 (Fig. 5A), thioredoxin-interacting protein (Txnip) (Fig. 5B), methionine sulfoxide reductase (Msr) (Fig. 5C), and endoplasmic reticulum resident protein 44 (ERp44) (Fig. 5D). The four interactions had average sequence identity of 58.32, 48.72, 36.93 and 58.29%, respectively, and average coverage of 72.63, 74.19, 73.97, and 84.65%, respectively to their template complexes. These template complexes have PDB IDs as 1 W89, 4LL4, 3PIN and 5XWM, respectively.

As shown in Fig. 5A, *A. veronii* Trx1 interacted with *O. niloticus* Trx2 via the 33th, 34th, 64th, 71th, 75-79th residues, and with *O. niloticus* Txnip via the 34th, 35th, 37th, 64th, 76-80th, 94-96th residues (Fig. 5B). Similarly, *A. veronii* Trx1 interacted with *O. niloticus* Msr through the 28-32th, 34-41th, 44th, 61th, 63th, 64th, 70-80th, 93th, 95-99th residues (Fig. 5C), and with *O. niloticus* ERp44 via the 35th, 37th, 39th, 40th, 74-79th, 97th residues (Fig. 5D). The Jaccard similarity between any two sets of interacting sites was as high as 0.23–0.40, indicating that *A. veronii* Trx1 has the tendency to bind to host proteins by the same interaction interface. Especially, the 76-79th residues of *A. veronii* Trx1 were involved in each interspecies PPI, which could be potential targets for the development of new antibacterial agents.

## Discussion

Although a growing number of aquatic animal diseases are reported to be caused by *A. veronii* in recent years, the molecular mechanisms underlying the disease remain largely unknown. In this study, intraspecies and interspecies PPI networks were constructed based on interolog and domain-based methods to help identify virulence factors that have not been validated experimentally and global understanding of the pathogenic mechanisms. To ensure the reliability of PPI networks, multiple strategies were adopted including strict limitation of the coverage of protein domains when using domain-based methods. For instance, two proteins were defined as a PPI only if all domains from the two proteins interacted with each other. Despite the fact that GO annotation, gene expression pattern, and subcellular localization information demonstrated the accuracy of PPI networks, there could still be false positives and false negatives in PPI networks. Proteins with higher degree or betweenness centrality play crucial roles in many cellular processes [32, 33], thus given that in this study, the virulence factors showed higher degree and betweenness centrality, indicating their functional importance. Among 195 virulence factors, the degree of 28 ranked in the top 10% of degree distribution (hubs). The average PCC between 27 (96.43%) virulence factors and their interacting proteins exceeded 0.30, meaning that these 27 virulence factors were party hubs and had the tendency to simultaneously interact with their partners. Seven and five out of the 27 virulence factors were involved in the biosynthesis of secondary metabolites and antibiotics, respectively (e.g., dihydrolipoamide dehydrogenase, pyruvate kinase, and glycerol-3-phosphate dehydrogenase), whereas the remaining virulence factors were involved in RNA degradation, cell cycle, amino acid metabolism, and so on.

Analysis of the interactions formed by the virulence factors revealed that they had the tendency to connect with each other and were enriched in two network modules. One of the modules consisted of 57 proteins, out of which 17 were virulence factors. Most of the virulence factors were annotated in the terms “phosphorelay signal transduction system” and “regulation of transcription, DNA-templated”, respectively. This observation was mainly because most of the proteins in the module were members of two-component regulatory systems, including KdpE, AdeR, ArcA, chemotaxis protein CheB, CheY, CpxR, OmpR, and PhoB. Two-component regulatory systems are important mediators of signal transduction and control bacterial virulence [34]. Thus, it is conceivable that the module is essential for the virulence of *A. veronii* and could serve as a target for future antimicrobial therapy. Nine out of the remaining 40 proteins directly interacted with the virulence factors. According to the “guilt-by-association” principle, i.e., interacting proteins tend to share similar biological function [35], the 9 proteins were likely to be virulence factors, although they were not predicted based on sequence homology. These 9 proteins included three copies of CheY, CreB, PhoB, CitB, CpxA, CheB and an unknown protein. Except CpxA which is a histidine kinase, the other 8 proteins are response regulators in two-component regulatory systems. It has been reported that many two-component regulatory systems, such as PhoP/PhoQ and EnvZ/OmpR, play important roles in virulence [36–39]. Thus, the 9 proteins could be potential drug targets.

Among the 57 proteins, 5 were histidine kinases, 31 response regulators, 6 diguanylate cyclases, 2 phosphodiesterases, and 10 unknown proteins. Based on the “guilt-by-association” principle, the 10 unknown proteins that interacted with the histidine kinases, response regulators, diguanylate cyclases or phosphodiesterases could also belong to one of the four types of proteins. Histidine kinase can sense environmental stimulus, while the corresponding response regulator mediates cellular response. These two proteins constitute the two-component regulatory system. Diguanylate cyclase synthesizes cyclic-diGMP and phosphodiesterase degrades cyclic-diGMP [40]. Cyclic-di-GMP as the second messenger transmits extracellular signals to intracellular environment. Since histidine kinases, response regulators, diguanylate cyclases, and phosphodiesterases co-exist in the same module, indicating that cyclic-di-GMP and two-component regulatory systems can work together to regulate *A. veronii* signal transduction. In *Xanthomonas campestris*, it has been demonstrated that cyclic-di-GMP binds to histidine kinase RavS to control two-component regulatory system RavS/RavR phosphotransferase [41], while in *Legionella pneumophila*, two-component system Lpg0278/Lpg0277 modules cyclic-diGMP metabolism [42].

Each virulence factor interacted with an average of 11 proteins in *O. niloticus*, which may be one of the reasons that pathogens with smaller genomes are able to overcome host with larger genomes. The *O. niloticus* proteins targeted by the virulence factors were mainly involved in “translation”, “cell redox homeostasis”, “protein folding”, “tricarboxylic acid cycle”, “glycolytic process”, “S-adenosylmethionine biosynthetic process”, “one-carbon metabolic process”, “ubiquitin-dependent protein catabolic process”, “ribosome biogenesis”, and “glycerol ether metabolic process”(Fisher’s exact test, *p* < 1.00 × 10^− 3^), implying that *A. veronii* could directly manipulate host metabolic processes, component organization, and homeostasis to achieve successful infection. Group A *Streptococcus* have been reported to deliver virulence factors into host cells during infection to modulate host metabolism by causing endoplasmic reticulum stress to induce asparagine formation. The formed asparagine can then be sensed by group A *Streptococcus* to increase its growth rate [43]. Thus, to block the nutritional source of pathogens, many host cells usually remain in a metabolically quiescent state during pathogen infection, which compels pathogens to reprogram host cell metabolism skewing it to obtain nutrients and energy [44]. In this process, virulence factors play an important role.

The findings from this study revealed that virulence factors of *A. veronii* probably hijack host pathways by mimicking host (*O. niloticus*) proteins, which is a common strategy used in pathogen-host interactions [45]. Virulence factors can mimic host global proteins, domains or short linear motifs to compete with endogenous interfaces of host [46]. In this study, only the mimicry of global proteins, which generated more tight interactions between virulence factors and host proteins were explored. Some of the virulence factors identified such as ClpP, could be used as preferred drug targets. In fact, many researchers have designed antibacterial drugs based on ClpP [30], with these results demonstrating potential application of virulence factors. Taken together, our results gives more insight into the potential application of virulence factors in antibacterial drugs development and treatment.

## Methods

### Construction of *A. veronii* PPI network

The interolog method was first used to infer the interactions between *A. veronii* proteins. Six organisms with large-scale experimental PPIs were selected as model organisms, including *A. thaliana*, *S. cerevisiae*, *C. elegans*, *D. melanogaster*, *E. coli* and *H. sapiens*. Protein sequences of these six model organisms were downloaded from the UniProt [47] database, and experimentally verified PPIs were collected from the BioGrid [48], IntAct [49], DIP [50] and MINT [51] databases. Additional PPIs of *A. thaliana* and *H. sapiens* were obtained from the TAIR [52] and HPRD [53] databases, respectively. Inparanoid Version 4.1 [54] was used to identify the orthologs between *A. veronii* and the six model organisms. A stringent threshold (inparalog score = 1.0) was set. Furthermore, the orthologs were analogized to predict *A. veronii* PPIs based on experimentally verified PPIs of the six model organisms.

The domain-based method was also used to infer *A. veronii* PPIs. Experimentally verified domain-domain interactions as templates were collected from the 3did [55] and iPfam [56] databases. Potential domains of *A. veronii* proteins were identified by PfamScan [57] (*e* ≤ 1.00 × 10^− 3^). Three strict standards were adopted to improve the prediction accuracy of *A. veronii* PPIs [25]. To start with, the protein domains with length coverage < 80% were filtered. Next, the total length of all domains in a protein was required to cover ≥40% of the protein. Finally, two proteins were defined as a PPI only if each domain in one protein interacted with each domain in the other protein. As a result, the *A. veronii* PPI network was constructed based on the *A. veronii* PPIs predicted by the interolog and domain-based methods.

### Assessment of *A. veronii* PPI network

Generally, two interacting proteins tend to have similar Gene Ontology (GO) annotations, similar gene expression patterns, and the same subcellular localization. To assess the reliability of the predicted *A. veronii* PPI network, 1000 random networks were generated by randomly rewiring edges of the *A. veronii* PPI network, while preserving the degree distribution. Semantic similarities of GO terms of interacting proteins in the *A. veronii* PPI network and random networks were calculated by the R package GOSemSim [58], including biological process, molecular function, and cellular component terms. Gene expression data of wild type as well as *argR*, *avrA*, *hfq*, *smpB* and tmRNA mutation in *A. veronii* from our previous studies (Supplementary Table S1) were used to evaluate the similarity of gene expression patterns of interacting proteins, which was quantified by absolute PCC. Subcellular localization of each protein was predicted by pLoc-mGneg [59], which was designed for Gram-negative bacteria and included eight subcellular localizations, i.e., cell inner membrane, cell outer membrane, cytoplasm, extracellular, fimbrium, flagellum, nucleoid and periplasm.

### Prediction of virulence factors

Virulence factors known to affect pathogen-host interactions were collected from the PHI-base database [60]. Sequence alignments were performed between *A. veronii* proteins and the known virulence factors by BLASTP. An *A. veronii* protein was predicted as potential virulence factor if the sequence identity was ≥40% and the coverage was ≥80% when aligned with a known virulence factor.

### Network characteristics analysis of virulence factors

The degree and betweenness centrality of virulence factors and other proteins in the *A. veronii* PPI network were calculated by the Cytoscape plugin NetworkAnalyzer [61], which is commonly used [62, 63]. The number of interactions between the virulence factors was counted. The same number of proteins as the virulence factors was randomly selected from the *A. veronii* PPI network and the number of interactions between the random proteins was also counted. This process was repeated 1000 times. The *A. veronii* PPI network was divided into modules by the Markov cluster algorithm (http://micans.org/mcl/). Only modules with at least five nodes were further analyzed. Fisher’s exact test was used to identify the modules enriched by the virulence factors and for annotation of the functions of modules.

### Prediction of virulence factor-*O. niloticus* protein interactions

A virulence factor has the potential to interact with *O. niloticus* proteins only if it is translocated into host cell. Thus, secreted virulence factors were first predicted by EffectiveDB [64], which integrates various tools to recognize bacterial secreted proteins. Sequences and function annotations of *O. niloticus* proteins were downloaded from the UniProt [47] database. Inparanoid Version 4.1 [54] was used to identify the orthologs between *O. niloticus* and the six model organisms (i.e., *A. thaliana*, *S. cerevisiae*, *C. elegans*, *D. melanogaster*, *E. coli* and *H. sapiens*), and potential domains of *O. niloticus* proteins were identified by PfamScan [57]. The interactions between the virulence factors and *O. niloticus* proteins were predicted based on experimentally verified PPIs of the six model organisms and experimentally verified domain-domain interactions. Fisher’s exact test was used to perform functional enrichment analysis of *O. niloticus* proteins.

### Structure modeling of virulence factor-*O.niloticus* protein interactions

Homologous template complexes of virulence factor-*O. niloticus* protein interactions were first searched in the PDB database [65] by BLASTP. Five criteria were considered [66–69]: (1) the alignment between each interacting protein and the template had ≥30% sequence identity and covered ≥40% of the interacting protein length; (2) the templates of two interacting proteins came from different chains of a protein complex structure in the PDB database and further constituted the template complex; (3) the template complex with resolution below 5 Å was prioritized; (4) X-ray structure as template complex was preferred over NMR structure; (5) average sequence identity of two interacting proteins with the template complex was given priority over average coverage, except when several template complexes had similar sequence identity, in which case the template complex with a higher coverage was preferred. Further, five models for each protein were generated using Modeller [70] based on the template. Among these, the model with the lowest Discrete Optimized Protein Energy (DOPE) score was regarded as the best structure of the protein after truncating unaligned residues at the N- and C-termini. Finally, the complex structure of two interacting proteins was inferred based on the template complex. The residues from two interacting proteins were defined as interacting sites if their shortest atomic distance was ≤4.0 Å. The Jaccard similarity for two sets of interacting sites was calculated by taking the number of their intersection divided by the number of their union.

## Supplementary Information

**Additional file 1: Table S1**. Gene expression data under different conditions in *Aeromonas veronii*

**Additional file 2: Table S2.** *Aeromonas veronii* protein-protein interaction network.

**Additional file 3: Table S3.** Interspecies protein-protein interaction network.

## Data Availability

The datasets generated during the current study are available at https://drive.google.com/drive/folders/18cHNUOSSJ5ugmFUL1_1QLVossf5ybYUY?usp=sharing and its supplementary information files.
